# Rapid idiosyncratic mechanisms of clinical resistance to KRAS G12C inhibition

**DOI:** 10.1172/JCI155523

**Published:** 2022-02-15

**Authors:** Yihsuan S. Tsai, Mark G. Woodcock, Salma H. Azam, Leigh B. Thorne, Krishna L. Kanchi, Joel S. Parker, Benjamin G. Vincent, Chad V. Pecot

**Affiliations:** 1 Lineberger Comprehensive Cancer Center,; 2 Division of Hematology/Oncology,; 3 Department of Pathology, and; 4 Department of Genetics, University of North Carolina at Chapel Hill, Chapel Hill, North Carolina, USA.; 5 Curriculum in Bioinformatics and Computational Biology and; 6 Computational Medicine Program, University of North Carolina School of Medicine, Chapel Hill, North Carolina, USA.; 7 Department of Microbiology and Immunology and; 8 Department of Medicine, University of North Carolina at Chapel Hill, Chapel Hill, North Carolina, USA.

**Keywords:** Genetics, Oncology, Adaptive immunity, Drug therapy, Molecular genetics

## Abstract

**BACKGROUND:**

The *KRAS* proto-oncogene is among the most frequently mutated genes in cancer, yet for 40 years it remained an elusive therapeutic target. Recently, allosteric inhibitors that covalently bind to KRAS G12C mutations have been approved for use in lung adenocarcinomas. Although responses are observed, they are often short-lived, thus making in-depth characterization of the mechanisms of resistance of paramount importance.

**METHODS:**

Here, we present a rapid-autopsy case of a patient who had a *KRAS^G12C^*-mutant lung adenocarcinoma who initially responded to a KRAS G12C inhibitor but then rapidly developed resistance. Using deep-RNA and whole-exome sequencing comparing pretreatment, posttreatment, and matched normal tissues, we uncover numerous mechanisms of resistance to direct KRAS inhibition.

**RESULTS:**

In addition to decreased KRAS G12C–mutant allele frequency in refractory tumors, we also found reactivation of the MAPK pathway despite no new mutations in *KRAS* or its downstream mediators. Tumor cell–intrinsic and non–cell autonomous mechanisms included increased complement activation, coagulation, and tumor angiogenesis, and several lines of evidence of immunologic evasion.

**CONCLUSION:**

Together, our findings reveal numerous mechanisms of resistance to current KRAS G12C inhibitors through enrichment of clonal populations, KRAS-independent downstream signaling, and diverse remodeling of the tumor microenvironment.

**FUNDING:**

Richard and Fran Duley, Jimmy and Kay Mann, the NIH, and the North Carolina Biotechnology Center.

## Graphical abstract

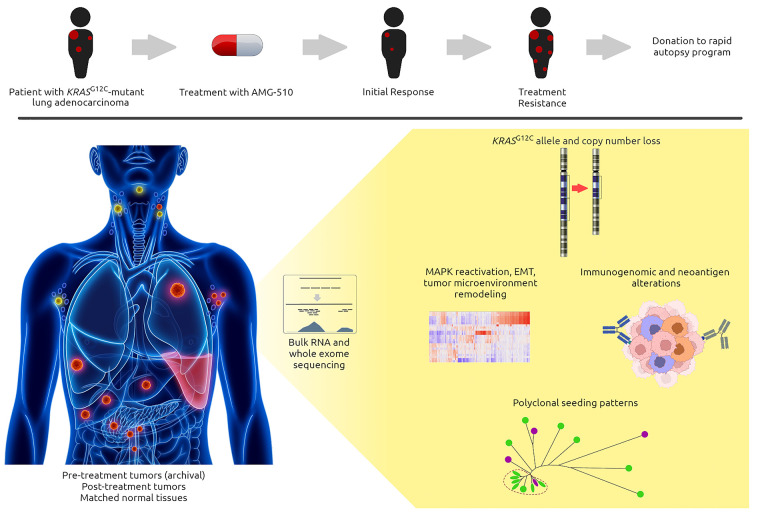

## Introduction

Constitutively activated, mutant *KRAS* continuously stimulates downstream effector signaling and promotes nearly all cancer hallmarks (1). Hotspot mutations that predominately occur in codons 12 and 13 result in defective KRAS GTPase activity, thus locking it into an active GTP-bound state. A remarkable breakthrough occurred when covalent allosteric inhibitors were discovered that could bind the cysteine residue within the switch II region of the KRAS G12C mutation, locking the protein in its inactive GDP-bound state (2, 3). More potent KRAS G12C inhibitors rapidly emerged, and recently expanded phase I trials with sotorasib (AMG510) and adagrasib (MRTX849) have demonstrated tumor response rates of approximately 30% to 40% in lung cancer and a safe toxicity profile (4–6). While encouraging, responses in the clinic are often short-lived, and mechanisms of clinical acquired resistance are beginning to emerge (7, 8). Here, we present a *KRAS^G12C^*-mutant lung adenocarcinoma patient who initially responded to AMG510 but then rapidly developed numerous mechanisms of resistance. Using deep-RNA and whole-exome sequencing (WES) of pre- and post-AMG510-treatment samples, we reveal diverse clonal populations that occurred through altered cell-intrinsic, tumor-microenvironment (TME), and immunologic remodeling mechanisms of resistance.

## Results

**Figure 1:**
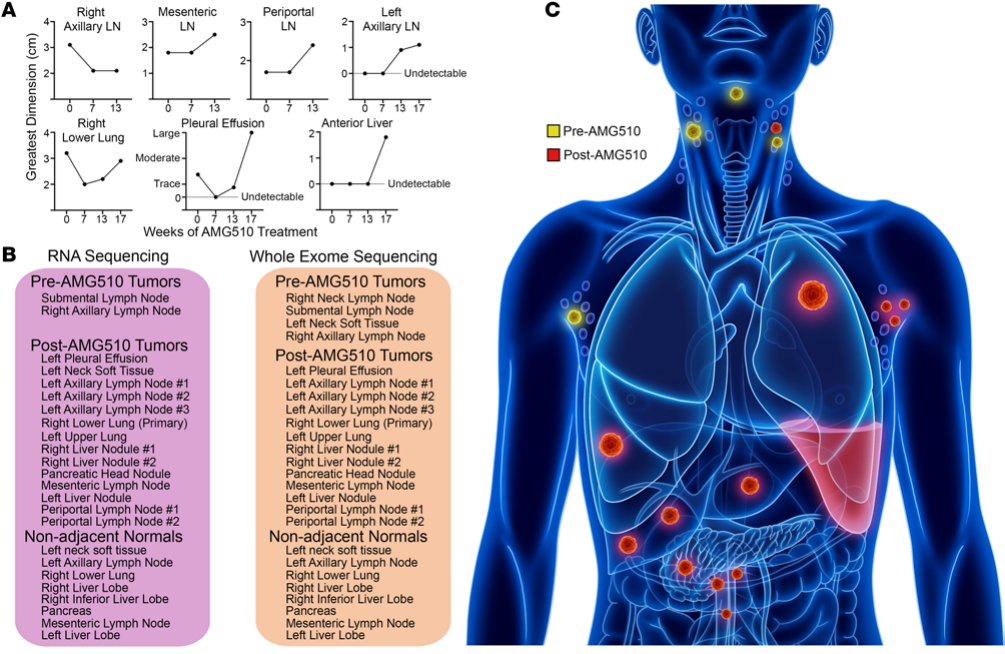
Rapid acquired resistance to AMG510. (**A**) Serial cross-sectional tumor dimensions while the patient received AMG510. (**B**) All samples were acquired either before the patient went on study (pre-AMG510) or during the rapid autopsy 6 weeks after stopping the drug (post-AMG510). All nonadjacent normal tissues were at least 1 cm from the tumor margin and confirmed by a pathologist to be free of tumor. (**C**) An illustration of the locations of tumors sampled for the study.

A 77-year-old male patient with prior smoking history was diagnosed with metastatic *KRAS^G12C^*-mutant lung adenocarcinoma and 80% expression of programmed death ligand 1 (PD-L1). The patient initially received chemotherapy and checkpoint inhibitor therapy targeting programmed cell death protein 1 (PD-1), and upon progression biopsies of several sites in the neck and the right axilla were obtained. The patient subsequently enrolled in a phase I clinical trial in which he received AMG510 (sotorasib) 960 mg twice daily dosing, and within a few days of starting treatment he clinically felt much improved with no significant side effects. Imaging 7 weeks after treatment initiation revealed that all lesions were stable or responding, and on average the measurable tumors had been reduced in size by approximately 35% (Figure 1A). However, scans at week 13 revealed that a few lesions had begun to grow, and at week 17, the study drug was discontinued due to rapid disease progression (Figure 1A). The patient died approximately 6 weeks later, and his body was donated to our rapid autopsy program in accordance with his wishes. Following pathologic confirmation of both tumor biopsies and matched nonadjacent normal tissues (>1 cm from tumor), RNA and DNA were extracted for RNA sequencing (RNA-Seq) and WES, respectively (Figure 1, B and C).

### Diverse and overlapping patterns of resistance.

**Figure 2:**
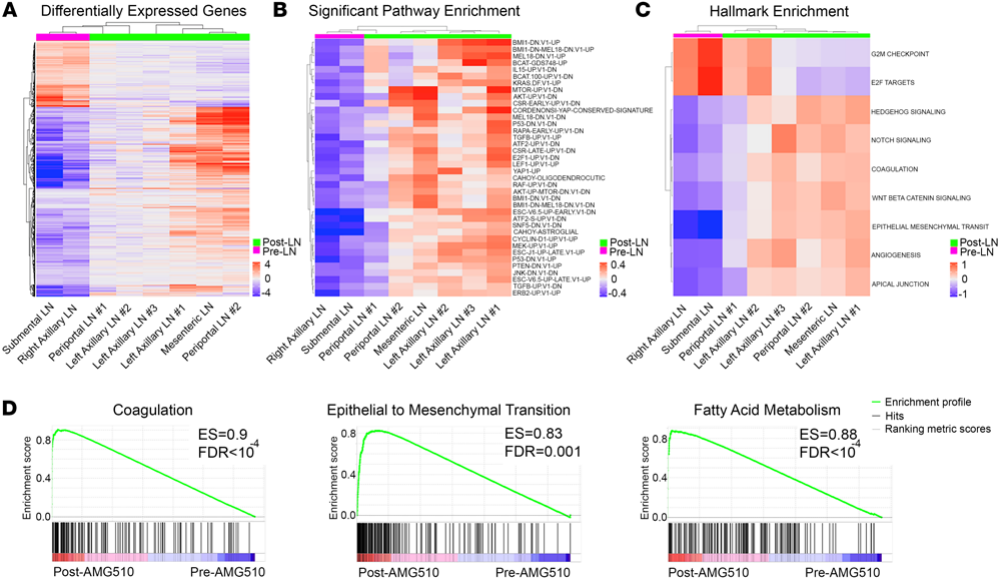
Dynamic adaptive cell-intrinsic reprograming and TME remodeling. (**A**) Nine hundred fifty differentially expressed genes with adjusted *P* values of less than 0.05 are shown as heatmaps, with 709 upregulated and 241 downregulated after AMG510 treatment. (**B**) Gene set analysis via GSVA using 189 MsigDB oncogenic signatures identified 38 oncogenic signatures (*P_adj_ <* 0.05) that were upregulated after AMG510 treatment. (**C**) GSVA analysis using 50 MsigDB Hallmark gene sets identified 9 signatures (*P_adj_ <* 0.05) that were differentially expressed, including upregulation in the Coagulation and EMT pathways. (**D**) GSEA for coagulation, EMT, and fatty acid metabolism.

Analysis of the transcriptome of 16 tumor and 8 nonadjacent normal samples revealed major differences between tissue origin types (Supplemental Figure 1; supplemental material available online with this article; https://doi.org/10.1172/JCI155523DS1), so we restricted the downstream gene and gene set expression analysis to pre- and post-AMG510 treatment lymph node (LN) tumors only to account for tissue-specific effects on gene expression in the TME. The transcriptome of the 2 pretreatment LN tumors was compared with 6 posttreatment LN tumors. A total of 950 genes were differentially expressed after AMG510 treatment (fold change > 1.5, *P_adj_ <* 0.05, and base mean > 10), compared with the pretreatment samples (Figure 2A). Seven hundred nine genes were upregulated and 241 genes were downregulated after AMG510 treatment. Gene set analysis was conducted using the gene set variation analysis (GSVA) method, and we computed gene set scores for the MSigDB oncogenic signature and cancer Hallmark gene set collections. Compared with pretreatment samples, nearly all the post-AMG510 treatment samples exhibited robust activation of the MAPK pathway, AKT, and mTOR signaling, with 38 significant oncogenic signatures (Figure 2B and Supplemental Tables 1–3). Consistent with genomic modeling of *KRAS* loss, we also found significant upregulation of pathways involving the transcriptional coactivator *YAP1* (9). Among the 9 significantly expressed Hallmark gene sets, 2 cell cycle gene sets were downregulated, G_2_/M checkpoint and E2F targets, consistent with dysregulated cell proliferation. The other 7 upregulated gene sets include activation of hedgehog, NOTCH, and WNT/β-catenin signaling as well as pathways for epithelial-mesenchymal transition (EMT) and tumor angiogenesis activation (Figure 2C). Nearly all these signatures were frequently observed in 6 out of the 7 posttreatment LN metastases (Figure 2, B and C), while the periportal LN sample number 1 (LN1) often resembled signaling patterns like pretreatment samples. We did observe varying degrees of reactivation of key pathways (notably mTOR, AKT, YAP1, and TGF-β) among posttreatment samples (Figure 2B). In parallel, gene set enrichment analysis (GSEA) revealed dramatic and noteworthy evidence of remodeling within the TME upon acquired resistance to AMG510. Indeed, TGF-β signaling and EMT signatures were reidentified, and strong evidence for complement activation, coagulation, and tumor angiogenesis were reminiscent of recalcitrant tumors mimicking chronic nonhealing wounds (10). Consistent with alterations in fuel sources, we observed marked increases in fatty and bile acid metabolism, as well as adipogenesis and myogenesis (Figure 2D and Supplemental Tables 4–6). Additionally, posttreatment tumors exhibited increased xenobiotic metabolism, suggesting tumors may be capable of reducing intracellular AMG510 levels. Using Ingenuity Pathway Analysis to evaluate the 950 differentially expressed genes, we found activated TGF-β signaling as the most significant upstream mediator of these diverse pathways of resistance (*P* < 2.1 × 10^–18^; Supplemental Figure 2 and Supplemental Table 7).

### Decreased KRAS G12C mutant allele frequency.

**Figure 3:**
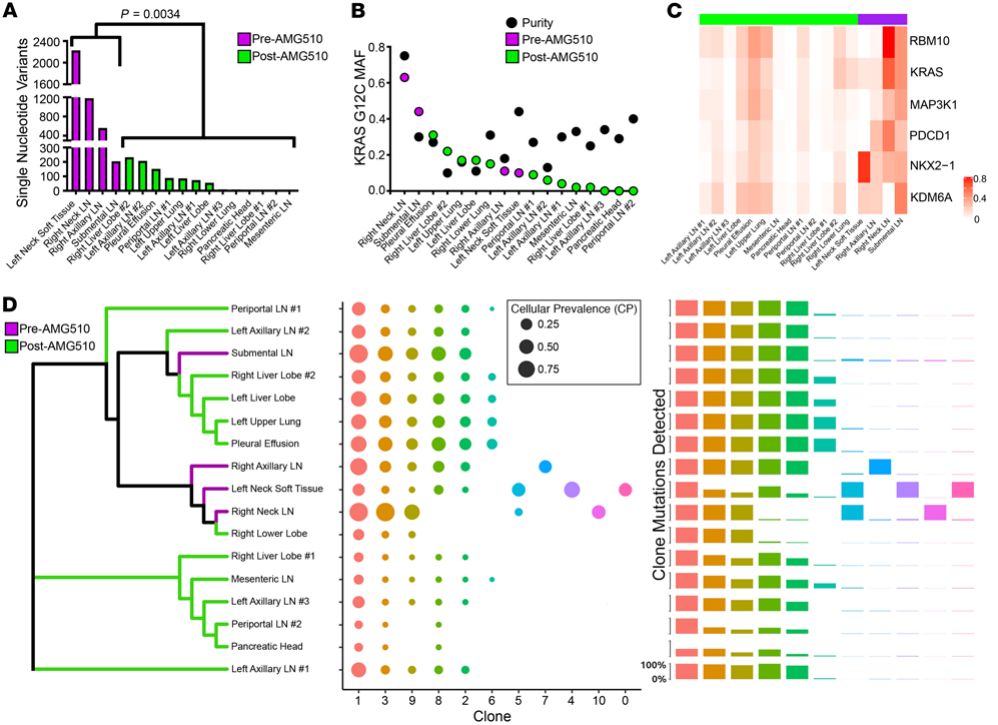
Loss of *KRAS^G12C^* variants and clonal phylogenetic analyses. (**A**) Total number of mutations detected in each tumor sample. A 2-sided Student’s *t* test was run to compare mean numbers of single nucleotide variants between pre- and post-AMG510 treatment. (**B**) *KRAS^G12C^* mutation allele frequency detected in each sample. Sequenza purity levels are shown for each sample in black. (**C**) Nonsynonymous mutations detected in 3 or more tumors with mutations expressed in RNA. (**D**) Representative phylogenetic trees showing potential clonal evolution. Pre- and posttreatment samples are colored in the branches as purple and green, respectively.

WES was performed on 4 pretreatment and 13 posttreatment tumors, as well as 8 nonadjacent normal samples (Figure 1). Note: The right neck LN was obtained at the time of diagnosis prior to any systemic therapy, while the submental LN, left neck soft tissue, and right axillary LN samples were obtained after chemotherapy and anti–PD-1 immune checkpoint therapy. Two nonadjacent normal samples with better sequencing quality were combined and used as matched normal for all tumor somatic mutation calling. Over 2,000 tumor single nucleotide variants (SNVs) were detected in the left neck soft tissue before treatment, and the other 3 pretreatment LN tumors also had significantly higher tumor variants called compared with all the posttreatment tumors (*P =* 0.0034; Figure 3A and Supplemental Figure 3). To identify mutations with potentially high biological impact, multiple filtering steps were applied to somatic mutation calling. First, only somatic mutations shared by at least 3 tumor samples were kept. Second, only variants with mutant allele frequency (MAF) greater than 5% were kept, ensuring a high-quality mutation calling. Finally, only variants expressed in any of the tumor RNA-Seq samples were used to further investigate potentially high biological impact. We cross-referenced these variants with a curated list of genes known to promote cancer progression mediated through mutations or copy number gain of function (11). Surprisingly, KRAS G12C mutations had a decreased MAF in most of the posttreatment samples (Figure 3B), suggesting a potential escape mechanism from AMG510 treatment. While the high number of SNVs called were associated with tumor purity calls (Pearson’s correlation = 0.51, *P =* 0.03), the MAF of KRAS G12C was not correlated with tumor purity (Pearson’s correlation = –0.017) in copy-neutral samples. Unlike recent studies that analyzed cell-free DNA (7, 8), we did not see new mutations that reactivate MAPK signaling and no new *KRAS* mutations were found in the posttreatment samples. We found a significant inverse Pearson’s correlation of KRAS G12C MAF with many of the resistance pathways, and a positive correlation with loss of G_2_/M cell cycle checkpoints (Supplemental Table 8), consistent with many of these being plausible escape mechanisms. Although 5 other mutations were found, none of them were new in the posttreatment samples and the MAF was often lower (Figure 3C), suggesting they were passenger mutations.

### Polyclonal seeding patterns from LN metastasis.

To evaluate clonal evolution among multiple metastases and between pre- and posttreatment samples, we estimated the cellular prevalence (CP) and subclonal structure using PyClone-IV (12, 13). Phylogenetic analysis using the CPs of subclones defined by PyClone-IV revealed there were multiple unique clones before AMG510 treatment (Figure 3D). The primary tumor (posttreatment) shared 3 subclones with the other 4 pretreatment samples and was in the same phylogenetic branch with 3 pretreatment samples. Five subclones (clones 5, 7, 4, 10, and 0) were unique to 3 pretreatment samples, and 4 of these subclones were private to one sample only. Most of the tumors showed multiclonal seeding patterns, except the primary tumor, which showed monoclonal seeding patterns (Supplemental Figure 4A). The pretreatment submental LN metastasis shared a common subclonal origin with 6 distant posttreatment metastases, suggesting it gave rise to these posttreatment sites (14). All tumors except the left neck soft tissue tumor (pretreatment) had clone 1 as a founding clone (Supplemental Figure 4A). In most tumors, clones 8 and 3 were derived from clone 1. Clone 3 then gave rise to subclones 2, 6, 9, and 10 in many tumors. Two variants from intron region of *RAI14* and coding region of *SPEF2* in cytogenetic band chr5p13 had the highest mean CP in the founding clone, clone 1, while clone 3 with KRAS G12C and 145 other mutations had the second highest mean CP (Supplemental Table 9). Fewer than 150 mutations were clustered within each shared subclone (Supplemental Figure 4B), while the 4 private clones had a minimum of 273 mutations (clone 7) and a maximum of over 3000 mutations in clone 0. Consistent with periportal LN1 having a divergent signature pattern from the periportal LN2 and mesenteric LN samples (Figure 2, B and C), our phylogenetic analyses also revealed that periportal LN2 and mesenteric LN likely originated from the same clone, unlike periportal LN1 (Figure 3D), indicating that even adjacent metastatic lesions may originate from different clonal populations and have divergent resistance mechanisms.

### Copy number loss of KRAS^G12C^ in resistant tumors.

Like our SNV analyses (Figure 3A), we found the posttreatment samples had fewer copy number variations (CNVs) (Supplemental Figure 5). The pretreatment submental LN metastasis had the lowest *KRAS^G12C^* CNV of any pretreatment sample (Supplemental Figure 6A), again demonstrating similarity with many posttreatment samples it likely gave rise to (Figure 3D). Tumor purity showed no significant association with *KRAS^G12C^* CNV (Supplemental Figure 6B; Pearson’s correlation = 0.12, *P =* 0.65), supporting the hypothesis that observed CNV changes are reflective of tumor biology in the posttreatment samples and not an artifact. When evaluating copy number changes among cancer-related gene loci (11), there were 15 genes with copy number gains in at least 3 tumor samples (Supplemental Figure 7). However, all of these occurred in the pretreatment samples, suggesting that independent of *KRAS^G12C^* copy number loss, other CNVs were unlikely mediators of acquired resistance.

### Immunogenomic and neoantigen remodeling.

**Figure 4:**
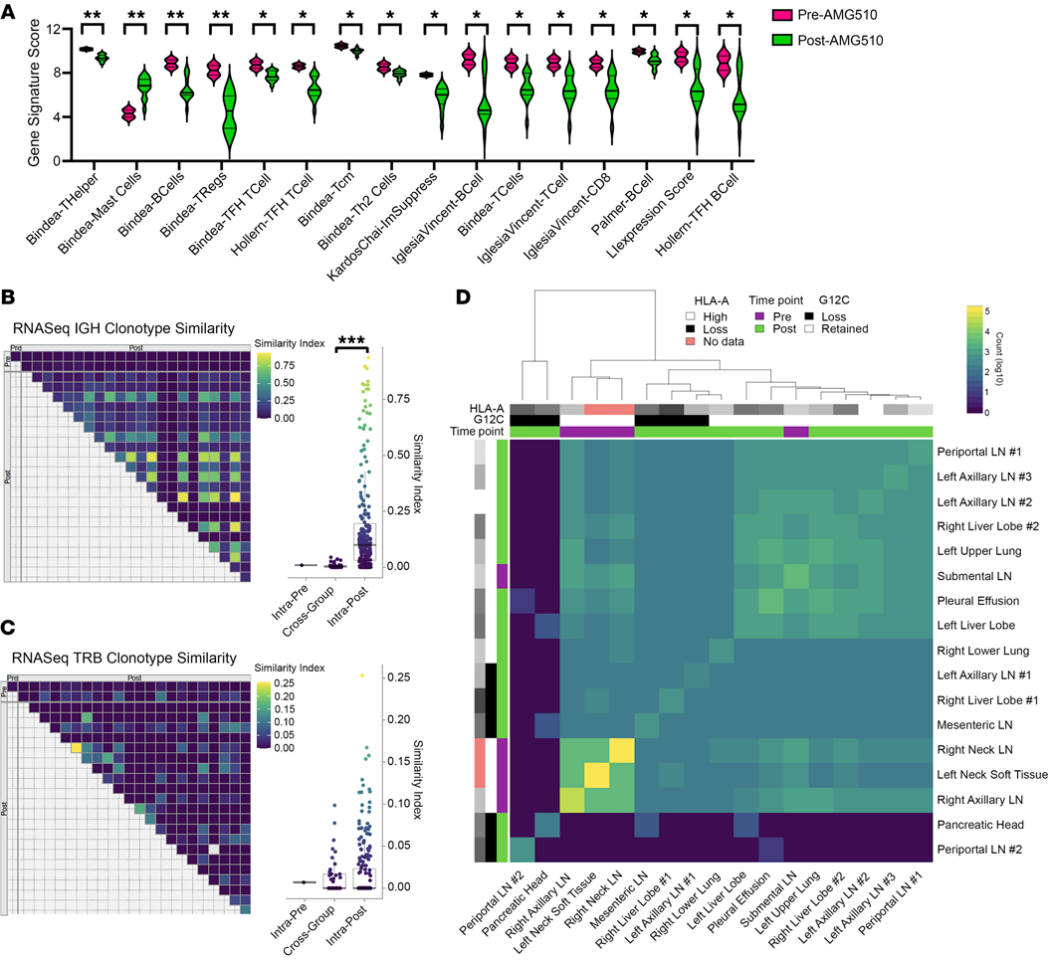
Tumor immunogenomic and neoantigen features. (**A**) Immune gene signatures by treatment time point, out of 40 tested signatures. (**B**) Tumor sample IgH repertoire comparison, by treatment time point. Horn’s modified Morisita overlap index. (**C**) Tumor sample T cell receptor β chain (TRB) repertoire comparison, by treatment time point. Horn’s modified Morisita overlap index. (**D**) Intersample predicted neoantigen count overlap between samples, log_10_ scale. Significance was assessed with the 2-sided Student’s *t* test. *P < 0.05, **P < 0.01, ***P < 0.001.

We next evaluated immunogenomic features to understand how the TME differed before versus after AMG510 therapy. We found that multiple immune gene signatures related to T and B cell function and activation were significantly downregulated in the posttreatment samples (Figure 4A). We also observed a significant and unexpected increase in the mast cell signature score in the posttreatment samples (Figure 4A). Expression in posttreatment samples (Supplemental Figure 8) was subdivided into 2 distinct groups: a small set of samples that resembled the pretreatment expression, and a larger set with more pronounced underexpression across most signatures. The 6 posttreatment LN samples were evenly divided between these 2 groups, indicating that nodal tissue was not the source of these differences. Among the B cell receptor (BCR) immunoglobulin heavy chains inferred from tumor RNA-Seq, there were significantly more overlapping repertoires between posttreatment samples as compared with between pre- and posttreatment samples (Figure 4B). Inferred T cell receptor (TCR) β chains exhibited more overlap between pre- and posttreatment samples, as well as between posttreatment samples, such that there was no significant difference comparing between these groups (Figure 4C). Posttreatment similarity scores were substantially higher for BCR IgH compared with TCR β chain clonotypes. These results suggest a potential posttreatment BCR repertoire focusing in some tumor types, perhaps in response to a restricted set of shared tumor antigens. The low number of pretreatment samples (*n =* 2) limits detection for repertoire overlap between solely pretreatment samples. Predicted neoantigens from tumor WES data demonstrated higher neoantigen burden in pretreatment compared with posttreatment samples, mirroring the SNV data (Figure 4D). These results are consistent with immune editing via elimination of a subset of tumor clones concurrent with AMG510 therapy; however, we cannot be certain that this did not reflect stochastic or differential direct effects of AMG510 on tumor subclones. When comparing tumor phylogenies and neoantigen remodeling patterns of the 4 pre-AMG510-treatment samples, we observed that the right neck LN sample (obtained prior to chemotherapy and immunotherapy) clustered tightly with the left neck soft tissue and right axillary LN samples (Figures 3D and 4D). In contrast, the submental LN (which was obtained at the same time as the left neck soft tissue and right axillary LN) gave rise to many of the post-AMG510-treatment metastases (Figure 3D) and differed in its neoantigen clustering (Figure 4D). Considering these findings, we speculate that chemotherapy and immunotherapy were not the main drivers of differences found in the pre- and post-AMG510-treatment samples. KRAS G12C neoantigens were predicted to weakly bind this patient’s HLA-A allele, and while samples with KRAS G12C loss tended to co-occur in clusters, HLA-A expression was relatively stable across all pre- and posttreatment samples (Figure 4D).

## Discussion

This is one of the first tissue-based molecular analyses to our knowledge that uncovers acquired mechanisms of clinical resistance to the novel KRAS G12C inhibitor class. Previously, another group analyzed serial cell-free DNA from the plasma of a patient being treated with MRTX849 and found numerous resistance mechanisms that converge on RAS-MAPK reactivation, including discovery of a novel *KRAS^Y96D^* mutation (8). Similarly, using cell-free DNA analyses in another cohort of 38 patients, investigators frequently found bypass mechanisms to reactivate MAPK signaling, including a variety of acquired *KRAS* mutations or activating downstream mutations (e.g., *BRAF* or *MAP2K1*; ref. 7), and one patient was found to have copy number gain of *KRAS^G12C^* (7). A key limitation of our study is the lack of samples obtained at the time of response to AMG510. Although outside the scope of this study, another limitation is our lack of validation of the potential mechanisms of resistance. Future work will need to rigorously evaluate these pathways using valid model systems. Ongoing efforts to obtain clinical samples throughout the course of treatment, as well as to establish patient-derived xenograft models will create invaluable resources to begin tackling these questions.

Use of in vitro and in vivo model systems has shed light on several cell-intrinsic and non–cell autonomous mechanisms of resistance, and were recently reviewed (15). Upon KRAS G12C inhibition, subpopulations of cancer cells that enter a quiescent state can stimulate a compensatory increase in active, GTP-bound KRAS G12C protein via EGFR and aurora kinase signaling. This adaptive response can occur within a matter of days, and results in growing drug-insensitive populations (16). Consistent with many of the resistant tumors in our case, several groups have shown that reactivation of the MAPK and/or PI3K/AKT/mTOR pathway induces varying degrees of resistance (4, 17). Evidence that EMT plays a role in de novo and acquired resistance to KRAS or downstream MAPK inhibition has also been demonstrated in several models (18–20), and EMT pathways were markedly enriched in nearly all of our patient’s refractory tumors. One of the more intriguing cell-intrinsic mechanisms of resistance in our study is the finding that tumors had reduced *KRAS^G12C^*-mutant allele frequency while maintaining “oncogene addiction” to the downstream pathways. However, unlike prior reports (7, 8), we found no evidence that other *KRAS* mutations or related MAPK or PI3K/AKT/mTOR signaling molecules were mutated, suggesting reactivation cues may come from upstream receptors (e.g., EGFR, HER2, c-MET; ref. 21) or from molecules such as TGF-β in the TME (19). Activated TGF-β signaling was the most significant upstream mediator of many overlapping resistance pathways, making it an attractive future area of study and potential therapeutic intervention. Another striking finding in our study is the dynamic and diverse patterns of remodeling within the TME, such as activation of angiogenesis and coagulation pathways, as well as alterations in fatty acid and xenobiotic metabolism. Unlike findings in the syngeneic CT26 colon cancer model that showed increased CD8^+^ T cells shortly after AMG510 treatment (5), our immunogenomic analyses revealed that AMG510-resistant tumors became immunologically cold and had significantly reduced adaptive immune cell populations. Interestingly, our findings that mast cell populations were increased in posttreatment samples is consistent with a previous connection demonstrated between KRAS-mediated EMT activation and mast cell recruitment (22). The neoantigen data are consistent with immune editing (23) in the context of AMG510 treatment; the number of predicted neoantigens was decreased in posttreatment samples, consistent with an active immune response eliminating neoantigen-expressing clones followed by escape with outgrowth of the resistant clone found initially in the submental LN. The mechanism of immune stimulation via KRAS G12C inhibition is unknown, although in a preclinical model, AMG510 treatment was associated with enhanced T cell tumor infiltration and generation of antitumor immunological memory (5). Thus, immune escape may be a key feature of AMG510 resistance to be explored in future studies.

Our study reveals the potential for dynamic and highly idiosyncratic mechanisms of acquired resistance to direct KRAS G12C inhibition that involve reactivation of KRAS-mediated signaling, metabolic reprograming, EMT activation, and diverse TME alterations including coagulation, angiogenesis, and immune escape pathways. Taken together, these results show that although development of KRAS inhibitors is a monumental and long-awaited success, a deeper understanding and eventual anticipation of these escape mechanisms will be paramount to best utilize these drugs’ full potential.

## Methods

### Nucleic acid extractions and sequencing.

Following pathologic review of all samples to confirm presence of tumor or normal tissues (in the case of nonadjacent matched normal, defined as >1 cm from the tumor margin), samples were submitted for nucleic acid isolation. Deidentified snap-frozen (SF) and formalin-fixed, paraffin-embedded (FFPE) tissue samples were sent to the UNC Lineberger Comprehensive Cancer Center Translational Genomics Lab for nucleic acid isolation using the Maxwell 16 MDx Instrument (Promega, AS3000). DNA was extracted from approximately 10 mg of SF tissue using the Maxwell 16 Tissue DNA Purification kit (Promega, AS1030) and from FFPE slides using the Maxwell 16 FFPE Plus LEV DNA Purification Kit (Promega, AS1135) following the manufacturer’s protocols (Promega, TM284 and TM349, respectively). RNA was extracted from approximately 10 mg of SF tissue using the Maxwell 16 LEV simplyRNA Tissue Kit (Promega, AS1280) and from FFPE slides using the Maxwell 16 LEV RNA FFPE Kit (Promega, AS1260) following the manufacturer’s protocols (Promega, TM351 and TM408, respectively). DNA and RNA quality was measured using a NanoDrop spectrophotometer (Thermo Fisher Scientific, ND-2000C) and a TapeStation 4200 (Agilent, G2991AA). DNA and RNA concentrations were quantified using a Qubit 3.0 fluorometer (Life Technologies, Q33216). RNA-Seq and WES were performed on an Illumina NovaSeq 6000. RNA libraries were stranded and sequenced via 150-bp paired-end reads. The RNA isolated from FFPE samples was first treated with a Ribo-Zero Magnetic Kit for rRNA removal (Illumina) prior to library preparation. WES was run on DNA samples following library preparation with an Agilent SureSelectXT2 All Exon V6 kit and performed at 200× coverage.

### RNA-Seq data analysis.

The fastq files were aligned to the GRCh38 human genome (GRCh38.d1.vd1fa from the National Cancer Institute’s Genomic Data Commons [GDC]) using STAR v2.4.2 (24) with parameters set as follows: –outSAMtype BAM Unsorted –quantMode TranscriptomeSAM. Salmon v0.1.19 (25) was used to quantify transcript abundance for each sample using the human transcriptome defined by Gencode (release 22). Gene level counts were summed across isoforms and genes with low counts (sum expression across all samples <5) were removed before the downstream analyses. We tested genes for differential expression in DESeq2 (26) (v1.22.2) in R (v3.5.1). Genes with an absolute value of fold-change (FC) greater than 1.50 and adjusted *P* value of less than 0.05 (*P* value obtained by Wald test and correction for multiple testing via Benjamini-Hochberg method) were defined as differentially expressed genes. Immune gene signature expression was calculated by the mean expression of each gene within that signature.

### GSEA.

We evaluated gene set enrichment using GSVA v1.30.0 (27) in R. GSVA estimates gene set scores by calculating an enrichment statistic for each gene set in every sample. The gene set score matrix was then used to test for differentially expressed gene sets using limma (moderated 2-sided *t* test; ref. 28) (v3.38.3) package in R (v3.5.1). Gene set collections were downloaded from the MSigDB website (29, 30) (version 6.2 database was used). We tested for C5 ontology gene set, C6 oncogenic signature gene sets, and Hallmark gene sets (31). GSEA was also carried out with downloaded GSEA (v4.1.0) software (www.broadinstitute.org/gsea). We first calculated gene scores as –log_10_(*P* value) × log_2_(fold change) for every gene and ranked them by the gene scores. The ranked gene list was then entered into the GSEA software using the GseaPreranked tool with the following parameters: scoring_scheme=weighted, norm=meandiv, mode=Max_probe, include_only_symbols=true, set_max=500, nperm=1000, collapse=No_Collapse. The same 3 gene set collections were used as described above.

### Variant calling.

We picked 2 normal samples (right liver normal and left neck soft normal tissue) with better sequencing quality and combined them for tumor somatic calling. The somatic variant–calling workflow is documented at https://sc.unc.edu/lbg/workflows/nextflow/somatic, and the “lbg_hg38_SureSelect_AllExonV6” profile was used. Briefly, both tumor and normal sequence reads were mapped to the GRCh38 human genome reference (GRCh38.d1.vd1.fa from GDC) with BWA v0.7.17 (32), and realigned together with ABRA2 v2.23 (33), and processed using biobambam2 (v2.0.87) and its bamsormadup tool (https://github.com/gt1/biobambam2). Quality control was implemented using the GATK/Picard (v4.1.7.0) tool (http://broadinstitute.github.io/picard/). Somatic variants were called by 3 callers: Strelka2 v2.9.10 (34), Cadabra v2.23 (33), and Mutect2 v4.1.7.0 (35). Somatic variants were merged into a single variant call file and then converted to MAF via the vcf2maf (v1.6.21) tool. This MAF file includes all variants that were PASS from any of the variant callers and a column with an additional filtering for variants passing all of any individual caller’s filters and for variants whose quality score exceeded a configurable threshold. In Figure 3A, we only counted variants with high/moderate impact (change coding) and that have MAF greater than 5%.

### Reinterrogation and expressed variants.

We performed computational reinterrogation to improve mutation calling sensitivity while keeping low false positives. First, we used high-quality somatic variants from initial calls described above (PASS in the 3 callers and MAF larger than 5%). All variants called from every tumor were then combined into a single file, and the resulting mutations were queried again in all tumors to rescue mutations that might have been missed during the first round of variant calling due to low allele frequency. All reinterrogated mutations shared by at least 2 tumors (nonprivate mutations) were then queried in the RNA-Seq tumor samples to check whether variants were expressed. The expressed variant calling workflow can be found at https://sc.unc.edu/lbg/workflows/nextflow/expressed_variants

### DNA tumor purity estimation and copy number calling.

We applied Sequenza (v3.0.0), an R package that uses DNA sequencing data to estimate tumor purity and ploidy, and to calculate allele-specific CNV (36). Using the sequenza-utils function of Sequenza, the GC content in sliding windows was calculated from the Hg38 genome reference file in FASTA format. Normal and tumor bams generated from somatic variants calling workflow, as described above, are the input files for sequenza-utils. The genomic positions with sufficient sequencing depth (by default, >20 reads total from tumor and normal specimens) were used to determine homozygous and heterozygous positions in the normal specimen and calculates the variant alleles and allelic frequency for the tumor specimen and saved as a tab-delimited seqz text file. Using the seqz text file, the Sequenza R package performed GC normalization and allele-specific segmentation using the Copynumber package. Then, the sequenza-fit model was applied to infer the purity and ploidy parameters and copy number profiles using the log(posterior probability) for all pairs of the candidates. The purity and ploidy values of the sample were estimated through Bayesian inference and the results expressed in a selected confidence region and 1-point estimate.

### Tumor clonal analysis.

PyClone-VI was applied to all 17 tumor DNA samples using the mutation calls after computational reinterrogation, as described above, and allele-specific copy number calls from Sequenza, as described above. A total of 6519 variants fulfilling the following criteria were used: (a) a depth of greater than 20 in all 17 samples, (b) a MAF of greater than 0.15 in any of the 17 samples, and (c) a valid Sequenza copy number call. The final cluster assignment of all the variants is shown in Supplemental Table 9.

### Phylogenetic tree construction.

Using the cellular prevalence of all subclones for each of the 17 tumors estimated from PyClone-VI, we calculated a pairwise distance between sample pairs via the Jensen-Shannon divergence using the R (v4.0.0) software package “phyloseq,” as previously described (14, 37). The distance matrices reflected how many mutations of each sample diverged from the other. The distance matrices were also used to construct a phylogenetic tree via the neighbor-joining algorithm (using the R software package “ape”), which produce rooted trees without using the distance.

### Neoantigen prediction.

HLA major and minor class I alleles were determined from RNA expression data using OptiType v1.3.1 (38) via the authors’ published Docker container in RNA mode (--rna, as per https://github.com/FRED-2/OptiType). Annotated variant transcripts were created using ANNOVAR v2019Oct24 (39), using their suggested SNP filters: the Exome Aggregation Consortium (ExAC) repository (last updated May 16, 2019) and ANNOVAR’s modified dbSNP list, avnsp147. Functional prediction and annotation was done using the suggested dbNSFP v3.0a (40) database. MHC binding affinities for 8-, 9-, and 10-mer peptides were calculated using netMHCpan v4.0a (41). NeoPredPipe v4.0 (42) was used to orchestrate the process of variant filtering, transcript annotation, and binding affinity calculations.

### Immune repertoire inference.

Immune chains were inferred from RNA-Seq FASTQ files using MiXCR v2.1.9 (43) and, following their recommended methods for multiplex-PCR data, paired-end reads were aligned in default mode, followed by contig assembly and export. Immune repertoire similarity was calculated from the inferred chains using Horn’s modified Morisita overlap index.

### Statistics.

Results for each group were compared using Student’s *t* test (for comparisons of 2 groups) and 2-way analysis of variance (ANOVA) (for multiple group comparisons). Multiple hypothesis testing correction of these statistical results was made using the FDR. A *P* value of less than 0.05 was deemed statistically significant.

### Study approval.

The patient provided consent prior to death for a rapid autopsy, in accordance with protocols of the UNC at Chapel Hill Office for Human Research Ethics and the US Department of Health and Human Services. This study was approved by the IRB of the University of North Carolina.

### Accession numbers and data sharing.

Sample information for RNA-Seq and DNA-Seq fastQ runs was uploaded to the NCBI’s database of Genotypes and Phenotypes (dbGAP accession number phs002734).

## Author contributions

YST, MGW, BGV, JSP, and CVP designed experiments, performed the investigation, and provided software. YST, MGW, SHA, LBT, and CVP curated data. YST, MGW, KLK, BGV, JSP, and CVP conducted formal analyses of the data. YST, MGW, and KLP validated the data. YST, MGW, and CVP generated figures and wrote the original draft of the manuscript, which was reviewed and edited by all the authors. CVP conceived the study, acquired funding, and provided resources. The co–first authors, YST and MGW, contributed to the vast majority of the experimental design and execution, and YST is listed first as they performed a larger amount of bioinformatic analyses.

## Supplementary Material

Supplemental data

ICMJE disclosure forms

Supplemental table 1

Supplemental table 2

Supplemental table 3

Supplemental table 4

Supplemental table 5

Supplemental table 6

Supplemental table 7

Supplemental table 8

Supplemental table 9

## Version 1 01/06/2022

In-Press Preview

## Version 2 02/15/2022

Electronic publication
