# Supplementary material for: Rapid idiosyncratic mechanisms of clinical resistance to KRAS G12C inhibition
Source: J Clin Invest. 2022 Feb 15;132(4):e155523. doi: 10.1172/JCI155523 (PMC8843735; doi:10.1172/JCI155523)
Supplement: Supplemental data [file jci-132-155523-s150.pdf]

Rapid Idiosyncratic Mechanisms of Clinical Resistance to KRAS G12C Inhibition

Yihsuan S. Tsai, Mark G. Woodcock, Salma H. Azam, Leigh B. Thorne, Krishna L. Kanchi, Joel S. Parker, Benjamin G. Vincent, Chad V. Pecot

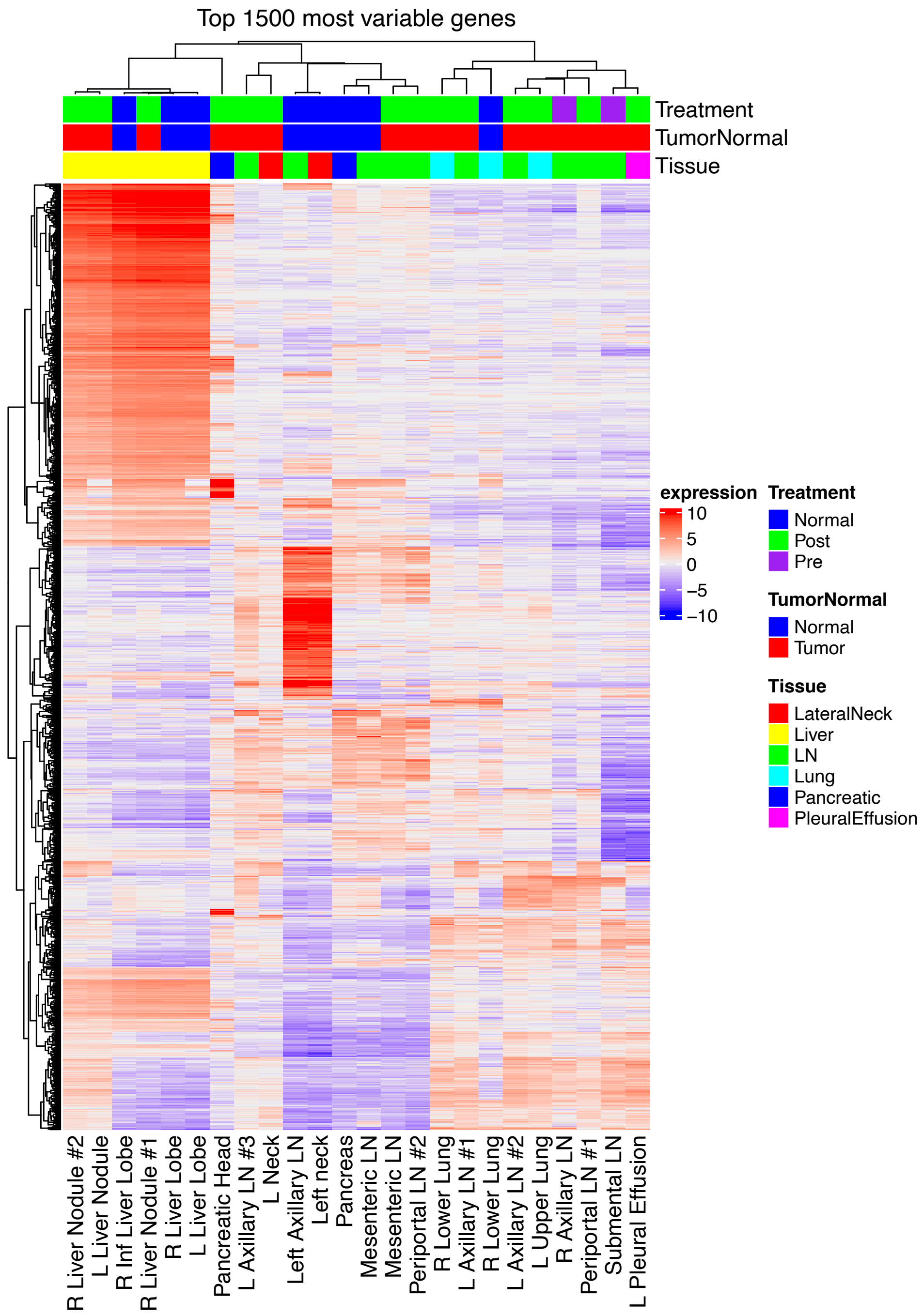

Supplementary Figure 1. Unsupervised heatmap of the top 1500 most variable genes. The top 1500 genes with the highest standard variation after VST (Variance Stabilizing Transformation) were used for samples clustering (distance: pearson, clustering method: average). VST gene expression were median-centered in the heatmap.

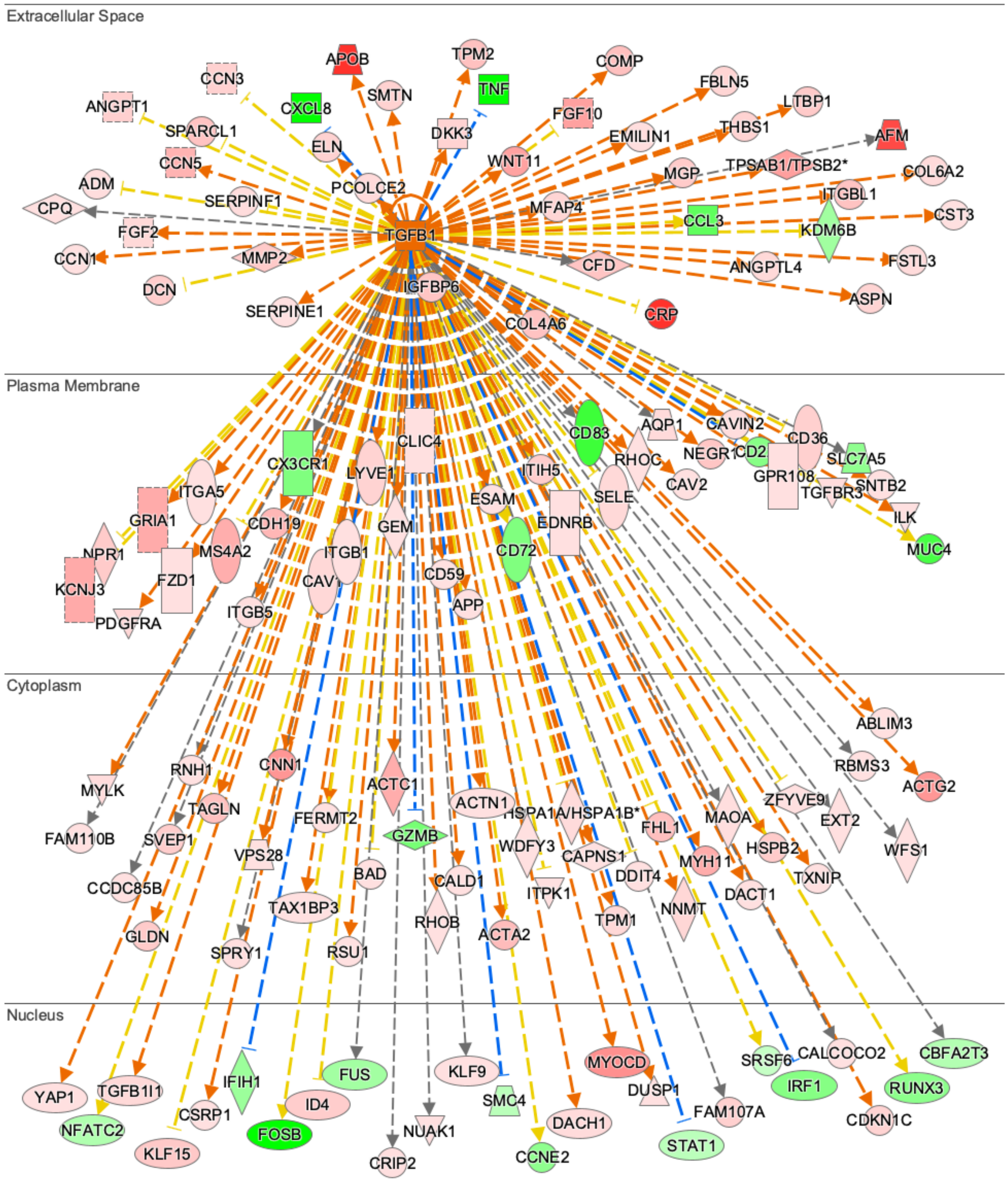

Supplementary Figure 2. Downstream TGFβ Signaling Network upon AMG510 Resistance. Ingenuity Pathway Analysis signaling network based on transcriptional programs derived from 950 differentially expressed genes (Figure 2) when comparing pre- and post-treatment lymph node samples.

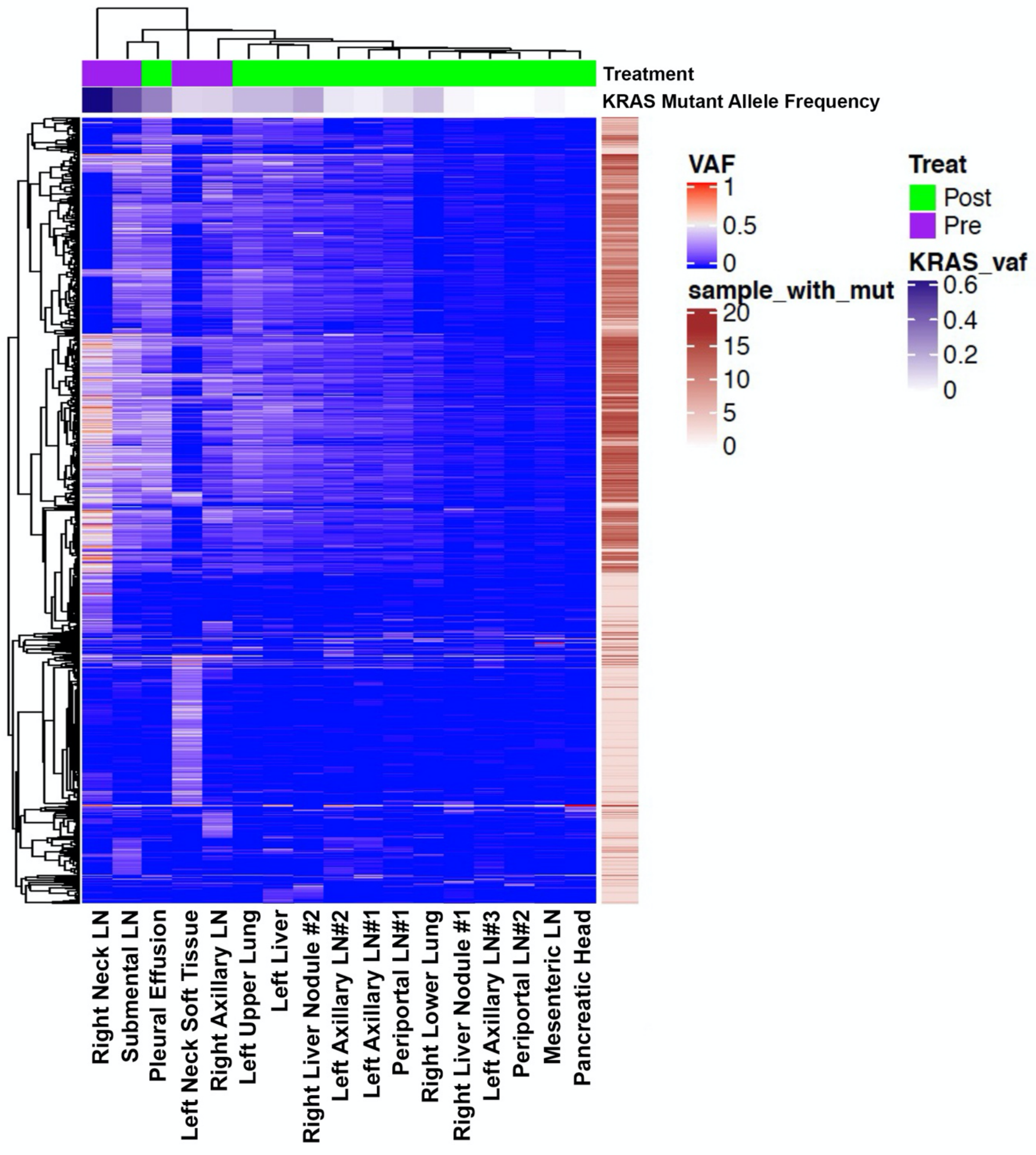

Supplementary Figure 3. Heatmap of VAF for 1283 somatic mutations shared by at least three tumors. Somatic mutations passed filtering criteria (methods) were combined across all tumors. Mutations shared by three or more tumor samples were shown in heatmap. The right color bar represents the number of tumors with each mutation (VAF > 0).

A

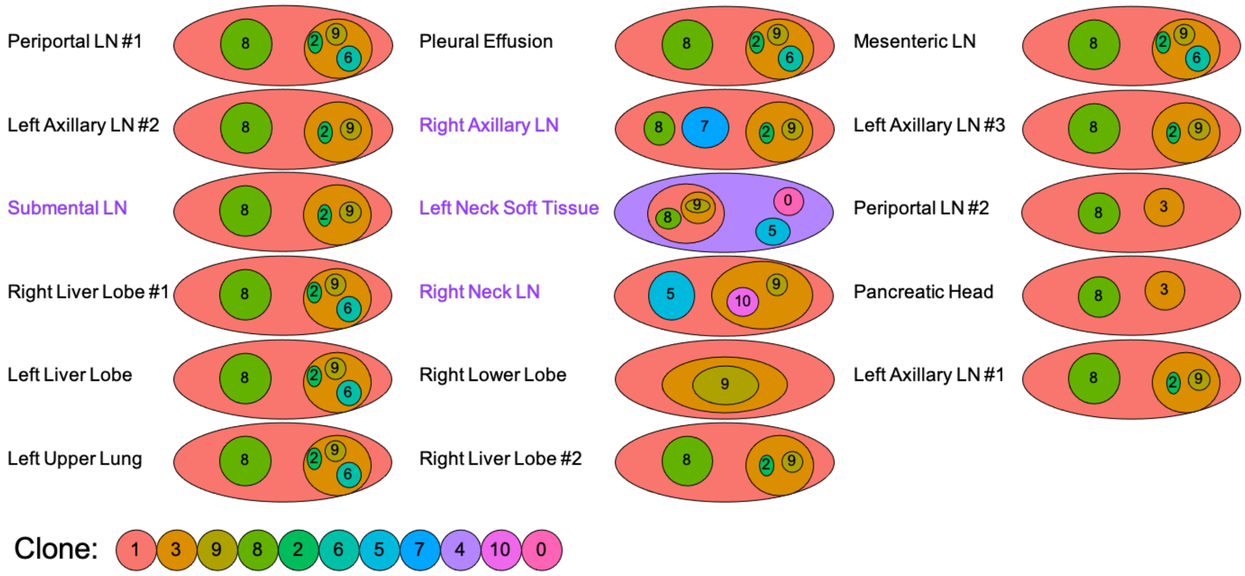

B

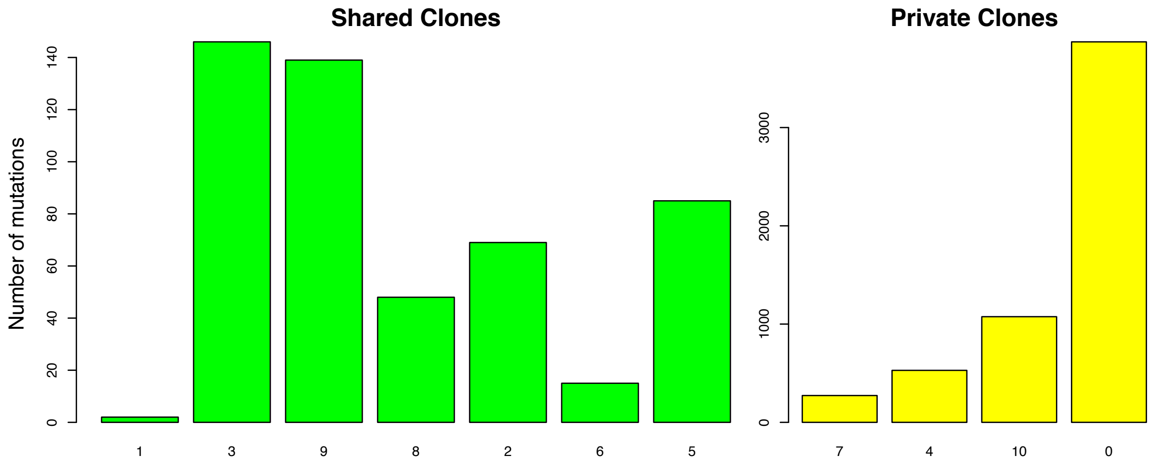

Supplementary Figure 4. Subclone seeding patterns in each tumor. Based on the cellular prevalence (CP), we depicted the clonal structure for each tumor. (**A**) The radius of each circle is not proportionate to the mean CP of that clone in each tumor. (**B**) The number of mutations in each subclone for shared clones (in green) and private clones (in yellow).

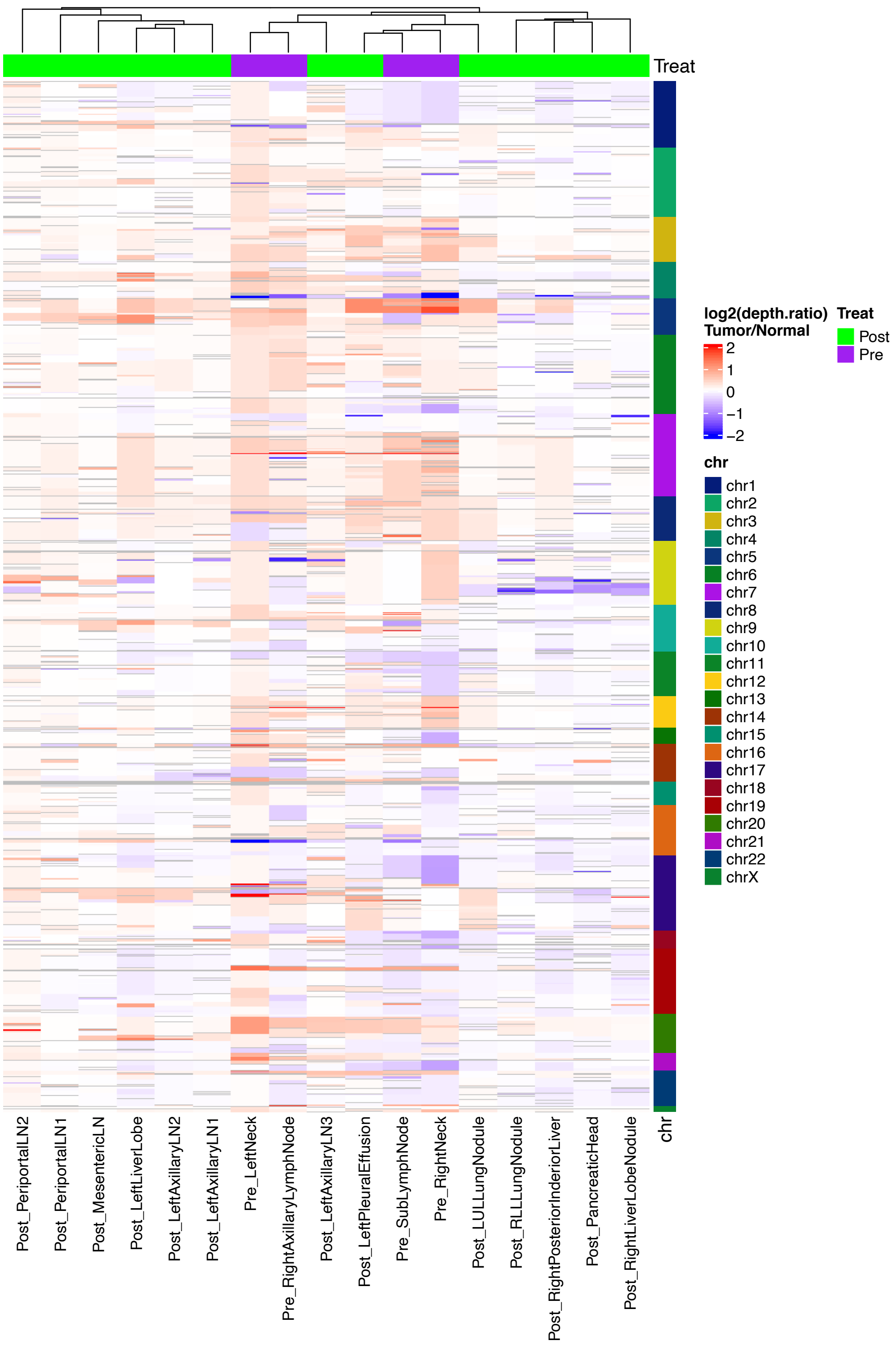

Supplementary Figure 5. Heatmap of copy number variations across the whole genome. Using the normal samples as reference, we applied Sequenza for the copy number calling for each tumor. The segments were then combined for all tumors. The right color bar represents which chromosome the loci located.

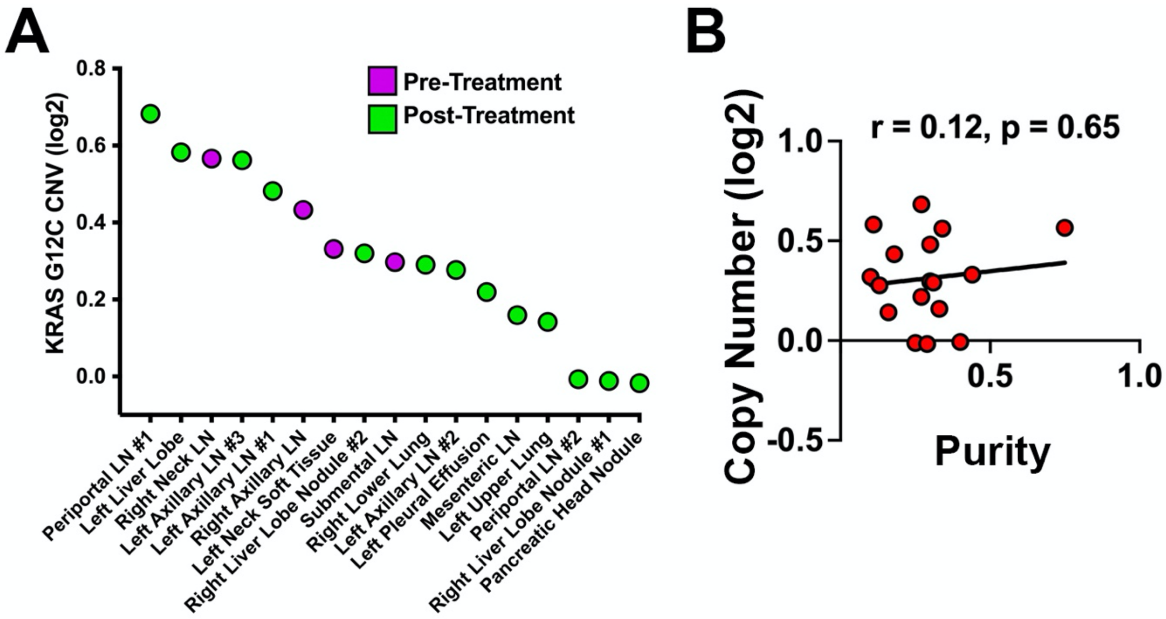

Supplementary Figure 6. KRAS G12C Copy number alterations and Purity Correlation. (**A**) KRAS G12C copy number variation (CNV) for pre- and post-treatment tumors. (**B**) Pearson correlation for KRAS G12C CNV (log2 scale) and Sequenza tumor purity.

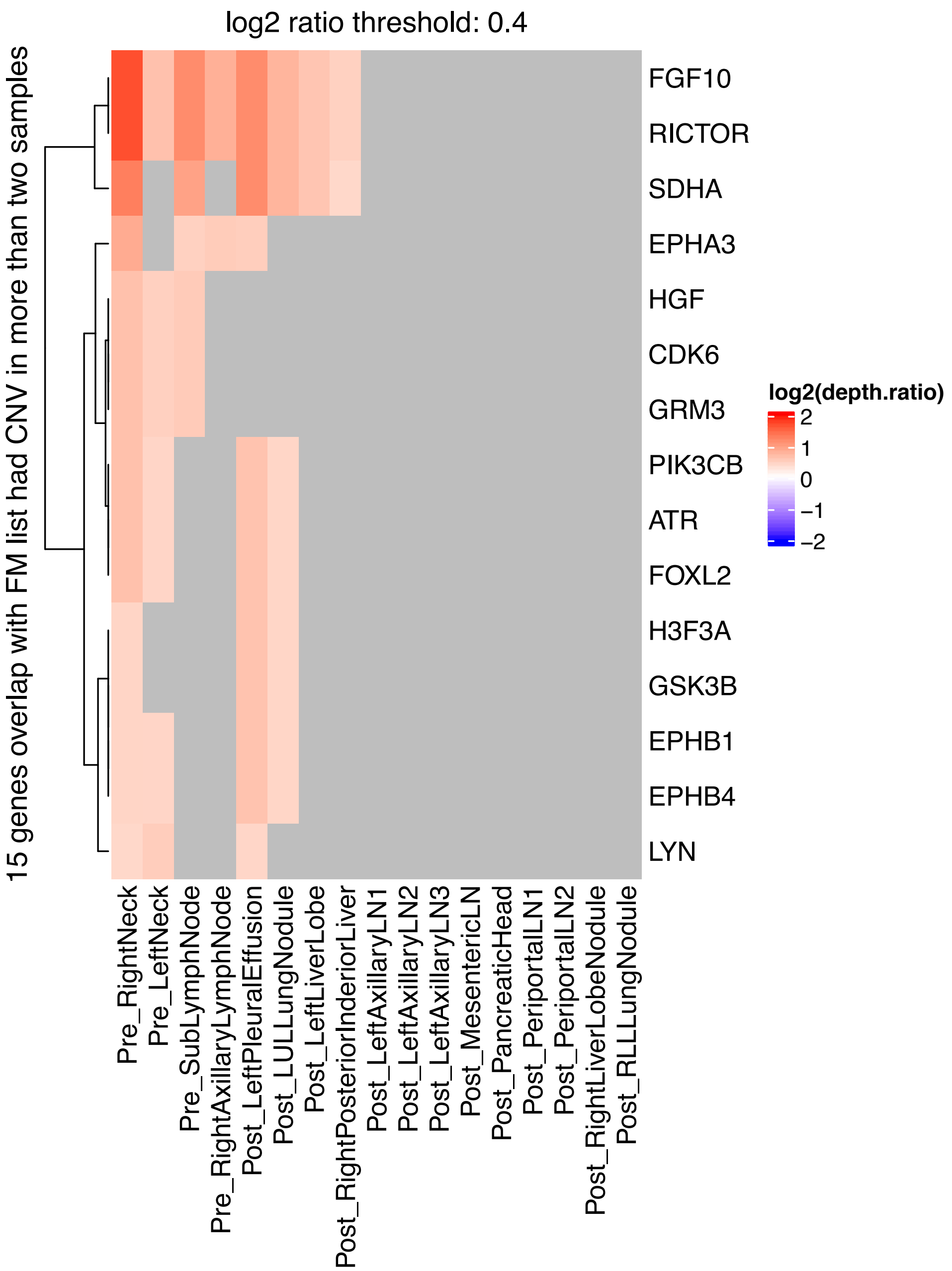

Supplementary Figure 7. Heatmap of copy number variations (CNV) for cancer-related genes. Using 0.4 as threshold for absolute value of log2 normalized depth ratio between tumor and normal, we identified 15 important cancer-related genes with copy number gains in at least three tumors. Ratios between -0.4 to 0.4 were grey out for visualization purpose.

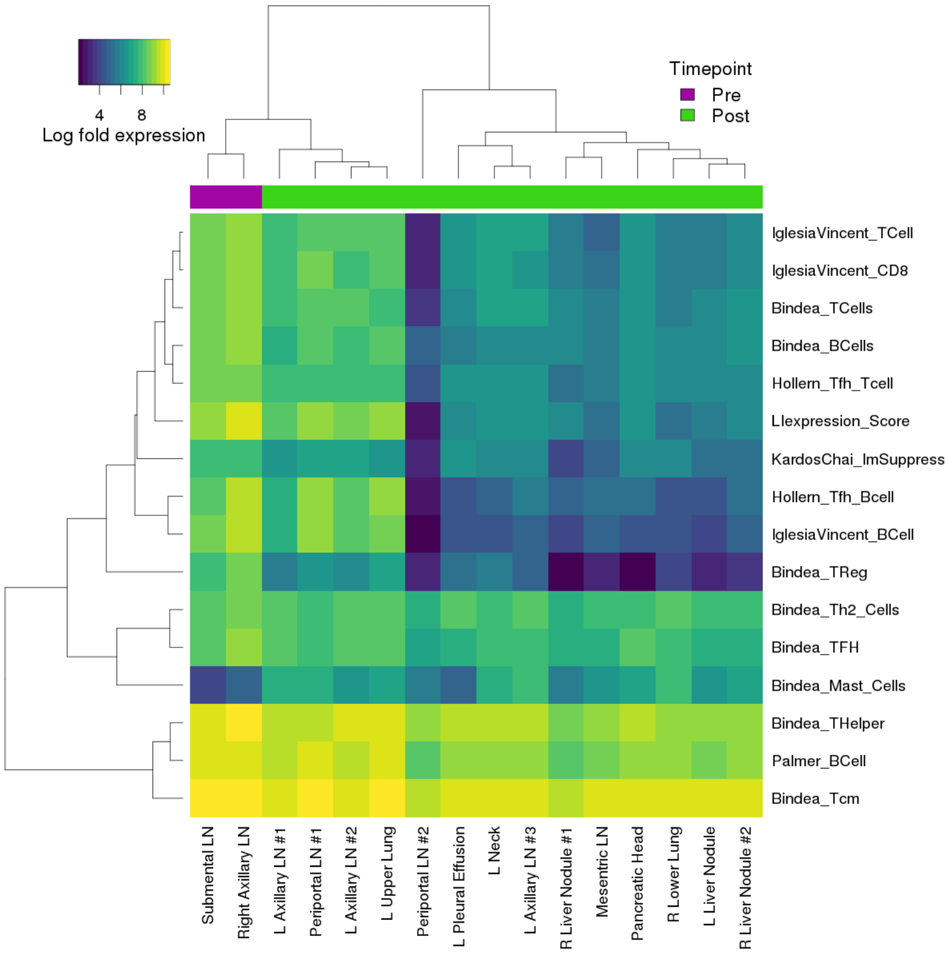

Supplementary Figure 8. Tumor immune gene signatures by sample.
